# The genome sequence of the confused eyebright, *Euphrasia confusa* Pugsley

**DOI:** 10.12688/wellcomeopenres.23301.1

**Published:** 2024-11-07

**Authors:** Alex D. Twyford, Royal Botanic Garden Edinburgh Genome Acquisition Lab, Darwin Tree of Life Barcoding collective, Wellcome Sanger Institute Tree of Life Management, Samples and Laboratory team, Wellcome Sanger Institute Scientific Operations: Sequencing Operations, Wellcome Sanger Institute Tree of Life Core Informatics team, Tree of Life Core Informatics collective, Darwin Tree of Life Consortium

**Affiliations:** 1 Royal Botanic Garden Edinburgh, Edinburgh, Scotland, UK; 2 The University of Edinburgh, Edinburgh, Scotland, UK

**Keywords:** Euphrasia confusa, eyebright, genome sequence, chromosomal, Lamiales

## Abstract

We present a genome assembly from a tetraploid specimen of the confused eyebright, *Euphrasia confusa* (Streptophyta; Magnoliopsida; Lamiales; Orobanchaceae). The genome sequence has a total length of 976.50 megabases. Most of the assembly is scaffolded into 22 chromosomal pseudomolecules, supporting the specimen being an allotetraploid (2 *n* = 4 *x* = 44). There are two mitochondrial genome scaffolds with lengths of 329.69 and 112.33 kilobases, and the plastid genome is 144.97 kilobases long.

## Species taxonomy

Eukaryota; Viridiplantae; Streptophyta; Streptophytina; Embryophyta; Tracheophyta; Euphyllophyta; Spermatophyta; Magnoliopsida; Mesangiospermae; eudicotyledons; Gunneridae; Pentapetalae; asterids; lamiids; Lamiales; Orobanchaceae; Rhinantheae; *Euphrasia*; *Euphrasia confusa* Pugsley (NCBI:txid475003).

## Background

Hemiparasitic plants – species that are green and photosynthesise but attach to and steal from other plant species – have captured the interest of biologists since the time of Darwin, and continue to be studied for their diverse and important roles in shaping natural ecosystems ( Twyford, 2018; Westwood *et al.*, 2010). Eyebrights ( *Euphrasia*) are one of the most species-rich genera of hemiparasites, with over 250 species. The genus is renowned for its diversity, with particularly notable variation in mating system (from selfing to outcrossing, ( French *et al.*, 2005) and ploidy (diploids to dodecaploids, Gussarova *et al*., 2008), as well as in its taxonomic complexity, with the c. 20 British species being extremely difficult to identify due to morphological similarity arising from recent speciation, frequent hybridisation, and extensive phenotypic plasticity ( Wang *et al.*, 2018).

The confused eyebright ( *Euphrasia confusa*) is a widespread annual plant species that is most commonly encountered in grazed pastures and grassy heathlands of northern and western Britain ( Stroh *et al.*, 2023). The species can typically be identified from the other British eyebrights, and in particular the closely related *E. arctica* and *E. nemorosa*, by a range of characters, such as its extensive (often basal) branching, its flexuous stems and branches, and its small flowers (5–7 mm) produced from a high node on the plant ( Metherell & Rumsey, 2018). Its taxonomic status, as its name implies, is far from certain, with population genetic analyses showing no clear differentiation from related taxa ( Ding *et al.*, 2023; French *et al.*, 2008). It hybridises with numerous other eyebright species producing an array of complex intermediate forms ( Stace *et al.*, 2015).

British eyebrights are either diploids or tetraploids, with *E. confusa* a tetraploid (2 *n* = 4 *x* = 44) ( Henniges *et al.*, 2022; Yeo, 1954), assuming a base number for species of *Euphrasia* is *x* = 11. Previous genomic analyses primarily based on short sequencing reads have shown British tetraploids are likely to be allotetraploids with divergent subgenomes, one coming from British diploids ( Becher *et al.*, 2020). However, the current tetraploid genome assemblies are highly fragmented.

The genome of the confused eyebright, *Euphrasia confusa*, was sequenced as part of the Darwin Tree of Life Project, a collaborative effort to sequence all named eukaryotic species in the Atlantic Archipelago of Britain and Ireland. Here we present a chromosomally complete genome sequence for *Euphrasia confusa*, based on a specimen from Whitten Pond, New Forest, UK. This is the first chromosome level genome assembly for the genus. This will be an important resource for future population genomic studies aimed at understanding species barriers, evolutionary processes and the genome dynamics of polyploids. It will also facilitate studies aimed at understanding the transition to plant parasitism, and the subsequent evolutionary consequences such as horizontal gene transfer

## Genome sequence report

**Figure 1.**
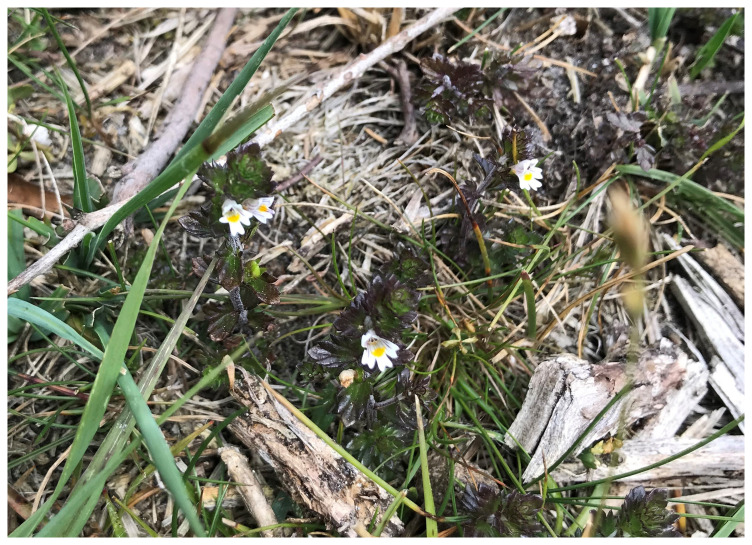
Photograph of the *Euphrasia confusa* plant that was sampled for genome sequencing.

The genome of a specimen of *Euphrasia confusa* ( Figure 1) was sequenced using Pacific Biosciences single-molecule HiFi long reads, 42.50 Gb (gigabases) from 4.54 million reads. The long-read coverage was estimated as 42-fold coverage, based on GenomeScope genome size estimation. Using flow cytometry, a previous study found the genome size (1C-value) of samples from this population range from 1.14–1.32 pg, equivalent to 1,115–1,285 Mb ( Becher *et al.*, 2021). Primary assembly contigs were scaffolded with chromosome conformation Hi-C data, which produced 271.26 Gb from 1,796.40 million reads. Specimen and sequencing details are summarised in Table 1.

**Table 1.** Specimen and sequencing data for *Euphrasia confusa*.

Project information			
**Study title**	*Euphrasia confusa* (eyebright)		
**Umbrella BioProject**	PRJEB61852		
**Species**	*Euphrasia confusa*		
**BioSample**	SAMEA7515968		
**NCBI taxonomy ID**	475003		
Specimen information			
**Technology**	**ToLID**	**BioSample ** **accession**	**Organism part**
**PacBio long read sequencing**	daEupConf1	SAMEA7516013	shoot
**Hi-C sequencing**	daEupConf3	SAMEA7516009	shoot
Sequencing information			
**Platform**	**Run accession**	**Read count**	**Base count (Gb)**
**Hi-C Illumina NovaSeq 6000**	ERR11439634	1.80e+09	271.26
**PacBio Sequel IIe**	ERR11413980	7.26e+05	8.07
**PacBio Sequel IIe**	ERR11413978	2.67e+06	23.98
**PacBio Sequel IIe**	ERR11413979	1.14e+06	10.45

**Figure 2.**
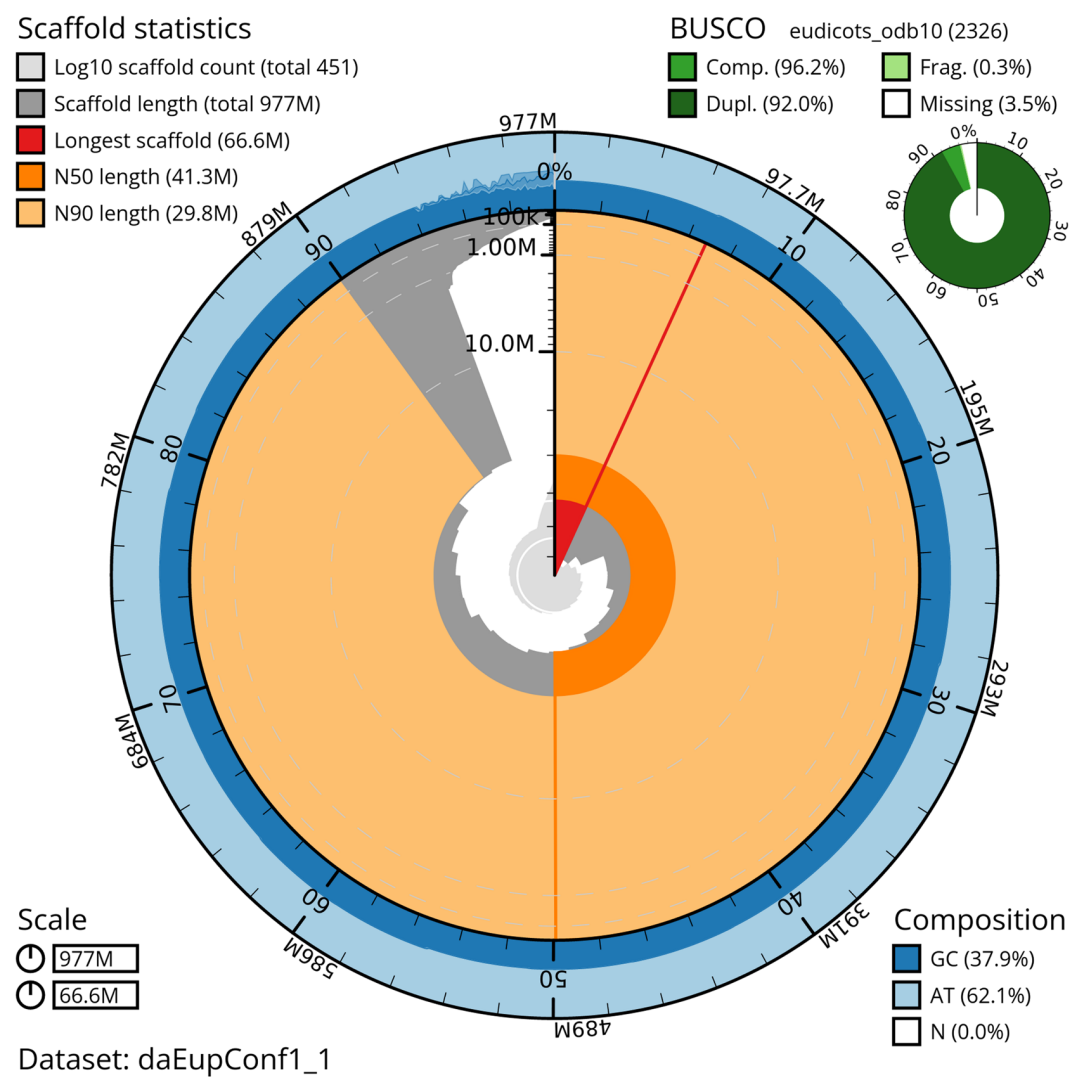
Genome assembly of *Euphrasia confusa*, daEupConf1.1: metrics. The BlobToolKit snail plot shows N50 metrics and BUSCO gene completeness. The main plot is divided into 1,000 size-ordered bins around the circumference with each bin representing 0.1% of the 977,066,659 bp assembly. The distribution of scaffold lengths is shown in dark grey with the plot radius scaled to the longest scaffold present in the assembly (66,608,978 bp, shown in red). Orange and pale-orange arcs show the N50 and N90 scaffold lengths (41,336,645 and 29,761,913 bp), respectively. The pale grey spiral shows the cumulative scaffold count on a log scale with white scale lines showing successive orders of magnitude. The blue and pale-blue area around the outside of the plot shows the distribution of GC, AT and N percentages in the same bins as the inner plot. A summary of complete, fragmented, duplicated and missing BUSCO genes in the eudicots_odb10 set is shown in the top right. An interactive version of this figure is available at https://blobtoolkit.genomehubs.org/view/daEupConf1_1/dataset/daEupConf1_1/snail.

**Figure 3.**
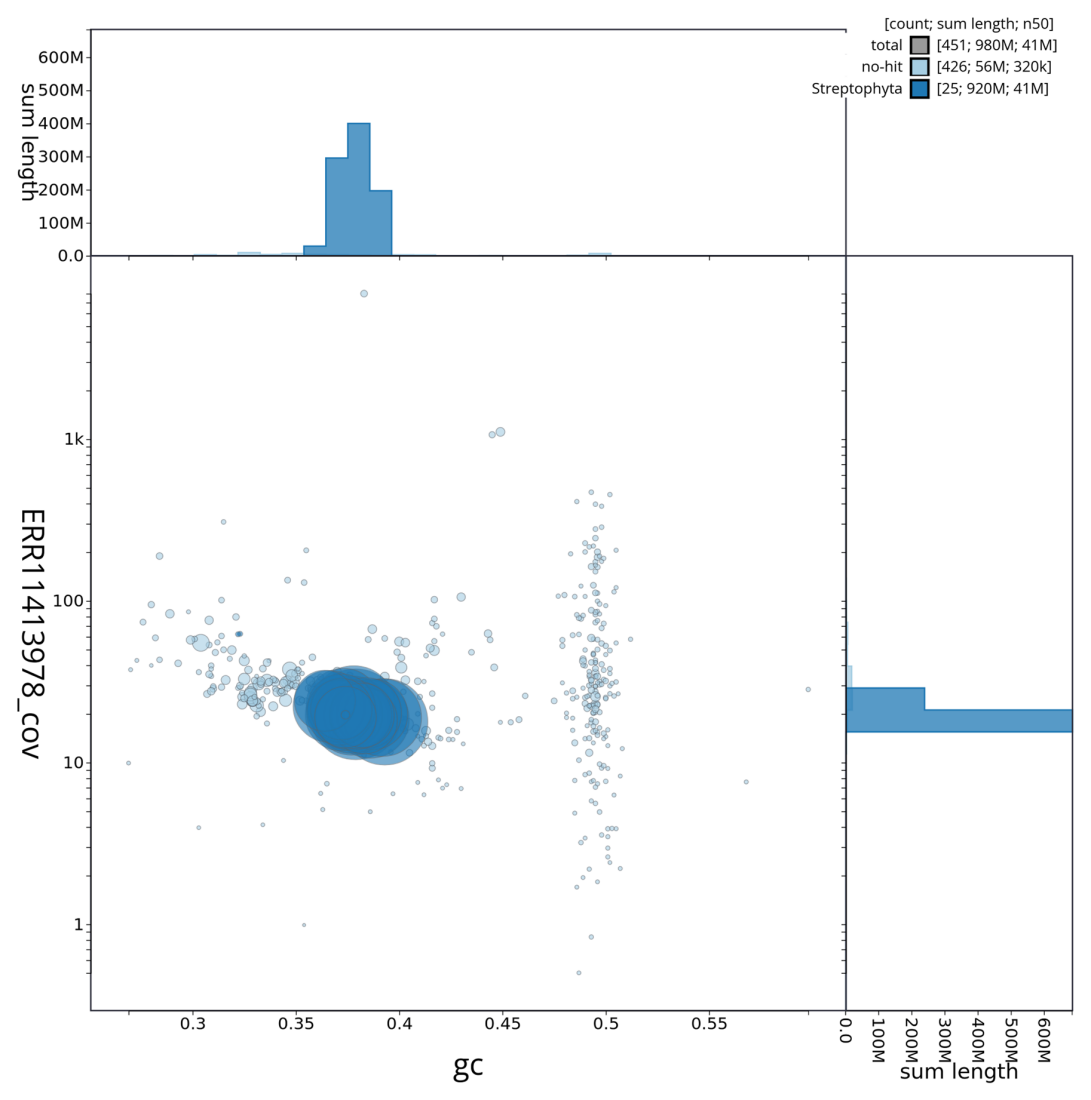
Genome assembly of *Euphrasia confusa*, daEupConf1.1: Blob plot of base coverage in ERR11413978 against GC proportion for sequences in assembly daEupConf1.1. Sequences are coloured by phylum. Circles are sized in proportion to sequence length. Histograms show the distribution of sequence length sum along each axis. An interactive version of this figure is available at https://blobtoolkit.genomehubs.org/view/daEupConf1_1/dataset/daEupConf1_1/blob.

**Figure 4.**
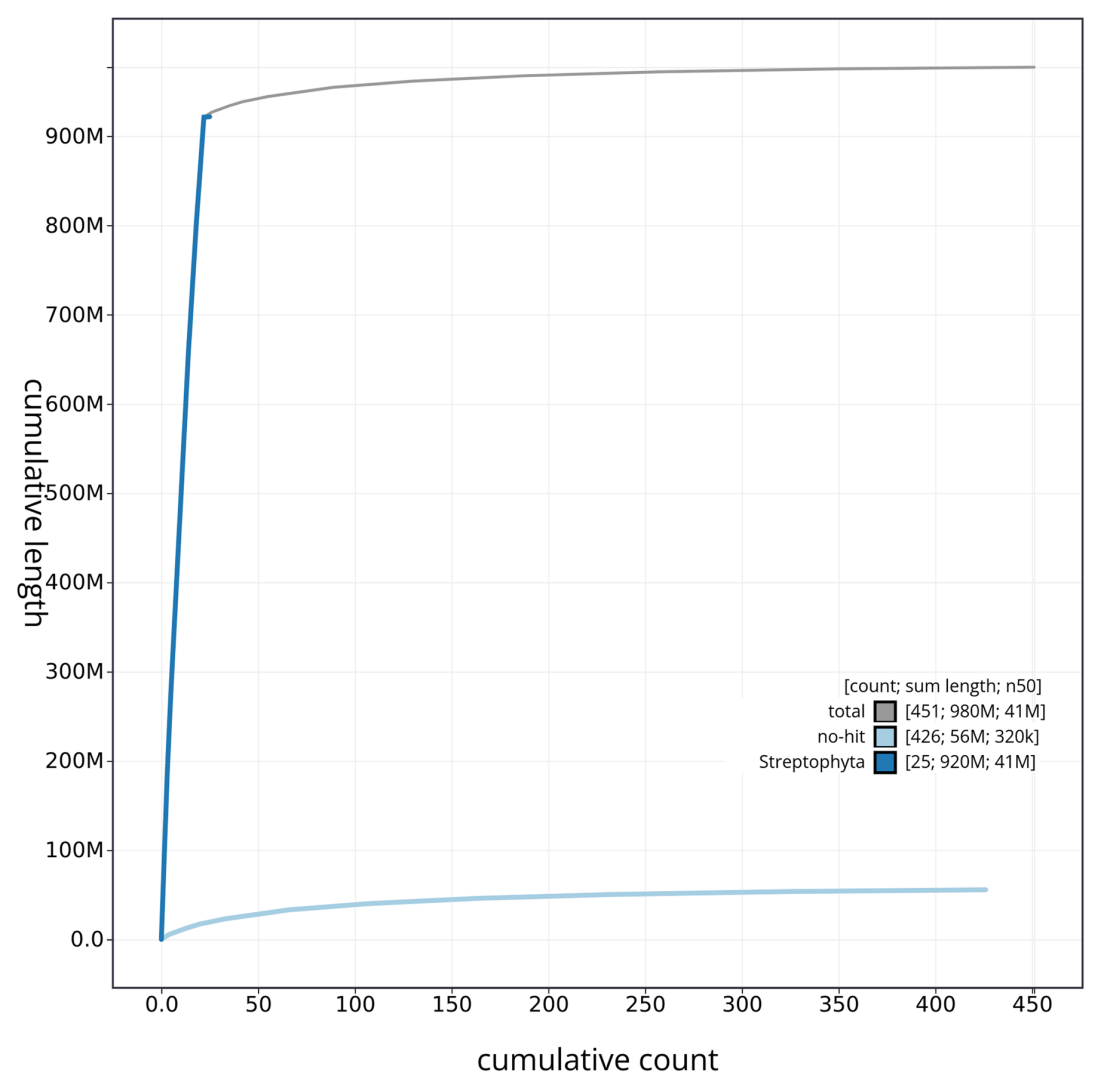
Genome assembly of *Euphrasia confusa* daEupConf1.1: BlobToolKit cumulative sequence plot. The grey line shows cumulative length for all sequences. Coloured lines show cumulative lengths of sequences assigned to each phylum using the buscogenes taxrule. An interactive version of this figure is available at https://blobtoolkit.genomehubs.org/view/daEupConf1_1/dataset/daEupConf1_1/cumulative.

**Figure 5.**
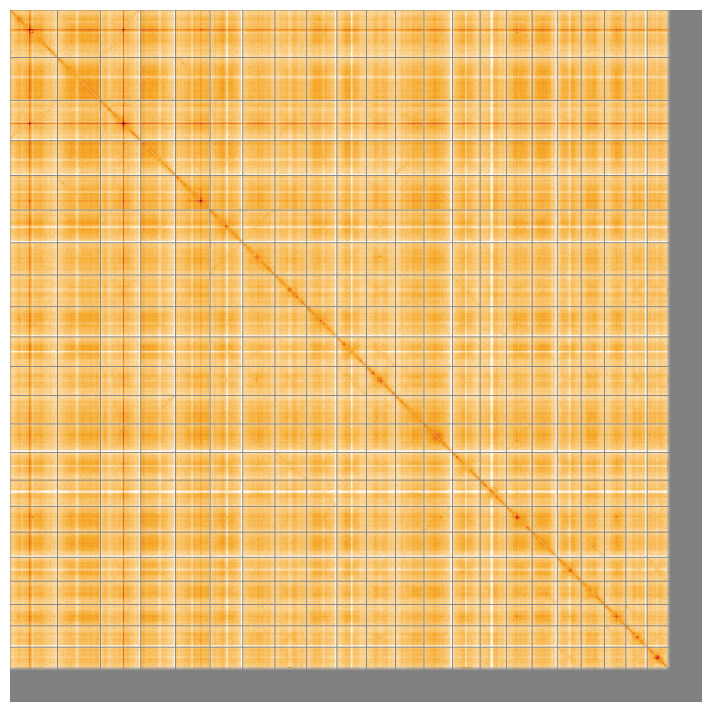
Genome assembly of *Euphrasia confusa*, daEupConf1.1: Hi-C contact map of the daEupConf1.1 assembly, visualised using HiGlass. Chromosomes are shown in order of size from left to right and top to bottom. An interactive version of this figure may be viewed at https://genome-note-higlass.tol.sanger.ac.uk/l/?d=e36EBlGAQnqAnaeKiSzb0w.

Manual assembly curation corrected 124 missing joins or mis-joins and two haplotypic duplications, reducing the scaffold number by 9.07%, and increasing the scaffold N50 by 23.53%. The final assembly has a total length of 976.50 Mb in 448 sequence scaffolds with a scaffold N50 of 41.3 Mb ( Table 2) with 192 gaps. The snail plot in Figure 2 provides a summary of the assembly statistics, while the distribution of assembly scaffolds on GC proportion and coverage is shown in Figure 3. The cumulative assembly plot in Figure 4 shows curves for subsets of scaffolds assigned to different phyla. Most (95.15%) of the assembly sequence was assigned to 22 chromosomal-level scaffolds. Chromosome-scale scaffolds confirmed by the Hi-C data are named in order of size ( Figure 5; Table 3). Given that there are 22 unique scaffolds, the data support that the specimen is an allotetraploid (2 *n* = 4 *x* = 44). Contigs corresponding to alternate haplotypes have also been deposited, however this is an incomplete representation of diversity due to the duplicate purging approach used for this genome. The mitochondrial and plastid genomes were also assembled and can be found as contigs within the multifasta file of the genome submission.

**Table 2.** Genome assembly data for *Euphrasia confusa*, daEupConf1.1.

Genome assembly		
Assembly name	daEupConf1.1	
Assembly accession	GCA_954870475.1	
*Accession of alternate haplotypes*	*GCA_954870545.1*	
Span (Mb)	976.50	
Number of contigs	643	
Number of scaffolds	448	
Longest scaffold (Mb)	66.61	
Assembly metrics *		*Benchmark*
Contig N50 length (Mb)	8.3	*≥1 Mb*
Scaffold N50 length (Mb)	41.3	*= chromosome N50*
Consensus quality (QV)	65.3	*≥ 40*
*k*-mer completeness	100.0%	*≥ 95%*
BUSCO **	C:96.2%[S:4.2%,D:92.0%], F:0.3%,M:3.5%,n:2,326	*S > 90%* D * < 5%*
Percentage of assembly mapped to chromosomes	95.15%	*≥ 90%*
Organelles	Mitochondrial genome: 329.69 and 112.33 kb; plastid genome: 144.97 kb	*localised* *homologous pairs*

* Assembly metric benchmarks are adapted from Rhie *et al.* (2021) and the Earth BioGenome Project Report on Assembly Standards September 2024. ** BUSCO scores based on the eudicots_odb10 BUSCO set using version 5.4.3. C = complete [S = single copy, D = duplicated], F = fragmented, M = missing, n = number of orthologues in comparison. A full set of BUSCO scores is available at https://blobtoolkit.genomehubs.org/view/daEupConf1_1/dataset/daEupConf1_1/busco.

**Table 3.** Chromosomal pseudomolecules in the genome assembly of *Euphrasia confusa, daEupConf1*.

INSDC accession	Name	Length (Mb)	GC%
OX940949.1	1	66.61	38.0
OX940950.1	2	60.05	39.5
OX940951.1	3	56.41	38.0
OX940952.1	4	48.52	38.5
OX940953.1	5	48.37	39.0
OX940954.1	6	45.97	37.5
OX940955.1	7	44.87	37.5
OX940956.1	8	44.5	37.5
OX940957.1	9	42.21	37.5
OX940958.1	10	41.34	37.0
OX940959.1	11	39.67	38.5
OX940960.1	12	40.86	36.5
OX940961.1	13	39.77	38.5
OX940962.1	14	38.89	37.5
OX940963.1	15	35.11	38.5
OX940964.1	16	36.34	38.0
OX940965.1	17	36.62	37.0
OX940966.1	18	33.37	37.0
OX940967.1	19	32.83	38.0
OX940968.1	20	29.76	37.0
OX940969.1	21	29.19	36.5
OX940970.1	22	29.85	37.5
OX940973.1	Pltd	0.14	38.5
OX940971.1	MT1	0.33	45.0
OX940972.1	MT2	0.11	44.5

The estimated Quality Value (QV) of the final assembly is 65.3 with *k*-mer completeness of 100.0%, and the assembly has a BUSCO v5.4.3 completeness of 96.2% (single = 4.2%, duplicated = 92.0%), using the eudicots_odb10 reference set ( *n* = 2,326).

Metadata for specimens, BOLD barcode results, spectra estimates, sequencing runs, contaminants and pre-curation assembly statistics are given at https://links.tol.sanger.ac.uk/species/475003.

## Methods

### Sample acquisition, DNA barcoding and genome size estimation

The specimens of *Euphrasia confusa* used for PacBio HiFi sequencing (specimen ID EDTOL00037, ToLID daEupConf1) and Hi-C sequencing (specimen ID EDTOL00033, ToLID daEupConf3) were collected from Whitten Pond, New Forest (latitude 50.8113, longitude –1.7159) on 2019-09-10. The specimens were collected and identified by Alex Twyford (University of Edinburgh) and kept on ice, then stored at –80 °C. The herbarium voucher associated with the sequenced plant is deposited in the herbarium of RBG Edinburgh (E).

The initial species identification was verified by an additional DNA barcoding process according to the framework developed by Twyford *et al*. (2024). Part of each plant specimen was preserved in silica gel desiccant. A DNA extraction from the dried plant was amplified by PCR for standard barcode markers, with the amplicons sequenced and compared to public sequence databases including GenBank and the Barcode of Life Database (BOLD). The barcode sequences for this specimen are openly available on BOLD ( Ratnasingham & Hebert, 2007). Following whole genome sequence generation, DNA barcodes were also used alongside the initial barcoding data for sample tracking through the genome production pipeline at the Wellcome Sanger Institute ( Twyford *et al.*, 2024). The standard operating procedures for the Darwin Tree of Life barcoding have been deposited on protocols.io ( Beasley *et al.*, 2023).

### Nucleic acid extraction

The workflow for high molecular weight (HMW) DNA extraction at the Wellcome Sanger Institute (WSI) Tree of Life Core Laboratory includes a sequence of procedures: sample preparation and homogenisation, DNA extraction, fragmentation and purification. Detailed protocols are available on protocols.io ( Denton *et al.*, 2023). The daEupConf1 sample was weighed and dissected on dry ice ( Jay *et al.*, 2023), and shoot tissue was cryogenically disrupted using the Covaris cryoPREP ^®^ Automated Dry Pulverizer ( Narváez-Gómez *et al.*, 2023). HMW DNA was extracted using the Automated Plant MagAttract v2 protocol ( Todorovic *et al.*, 2023). HMW DNA was sheared into an average fragment size of 12–20 kb in a Megaruptor 3 system ( Bates *et al.*, 2023). Sheared DNA was purified by solid-phase reversible immobilisation, using of AMPure PB beads to eliminate shorter fragments and concentrate the DNA ( Strickland *et al.*, 2023). The concentration of the sheared and purified DNA was assessed using a Nanodrop spectrophotometer and Qubit Fluorometer and Qubit dsDNA High Sensitivity Assay kit. Fragment size distribution was evaluated by running the sample on the FemtoPulse system.

### Hi-C preparation

Hi-C data were generated from shoot tissue of the daEupConf3 sample at the WSI Scientific Operations core, using the Arima-HiC v2 kit. Tissue was finely ground using cryoPREP, and then subjected to nuclei isolation using a modified protocol of the Qiagen QProteome Kit. After isolation, the nuclei were fixed, and the DNA crosslinked using a 37% formaldehyde solution. The crosslinked DNA was then digested using the restriction enzyme master mix. The 5’-overhangs were then filled in and labelled with biotinylated nucleotides and proximally ligated. An overnight incubation was carried out for enzymes to digest remaining proteins and for crosslinks to reverse. A clean up was performed with SPRIselect beads prior to library preparation. DNA concentration was quantified using the Qubit Fluorometer v2.0 and Qubit HS Assay Kit according to the manufacturer’s instructions.

### Library preparation and sequencing

Library preparation and sequencing were performed at the WSI Scientific Operations core. Pacific Biosciences HiFi circular consensus DNA sequencing libraries were prepared using the PacBio Express Template Preparation Kit v2.0 (Pacific Biosciences, California, USA) as per the manufacturer's instructions. The kit includes the reagents required for removal of single-strand overhangs, DNA damage repair, end repair/A-tailing, adapter ligation, and nuclease treatment. Library preparation also included a library purification step using AMPure PB beads (Pacific Biosciences, California, USA) and size selection step to remove templates shorter than 3 kb using AMPure PB modified SPRI. DNA concentration was quantified using the Qubit Fluorometer v2.0 and Qubit HS Assay Kit and the final library fragment size analysis was carried out using the Agilent Femto Pulse Automated Pulsed Field CE Instrument and gDNA 165kb gDNA and 55kb BAC analysis kit. Samples were sequenced using the Sequel IIe system (Pacific Biosciences, California, USA). The concentration of the library loaded onto the Sequel IIe was in the range 40–135 pM. The SMRT link software, a PacBio web-based end-to-end workflow manager, was used to set-up and monitor the run, as well as perform primary and secondary analysis of the data upon completion.

For Hi-C library preparation, DNA was fragmented to a size of 400 to 600 bp using a Covaris E220 sonicator. The DNA was then enriched, barcoded, and amplified using the NEBNext Ultra II DNA Library Prep Kit, following manufacturers’ instructions. The Hi-C sequencing was performed using paired-end sequencing with a read length of 150 bp on an Illumina NovaSeq 6000.

### Genome assembly, curation and evaluation

***Assembly***

The original assembly of HiFi reads was performed using Hifiasm ( Cheng *et al.*, 2021) with the --primary option. Hi-C reads were further mapped with bwa-mem2 ( Vasimuddin *et al.*, 2019) to the primary contigs, which were further scaffolded using the provided Hi-C data ( Rao *et al.*, 2014) in YaHS ( Zhou *et al.*, 2023) using the --break option. Scaffolded assemblies were evaluated using Gfastats ( Formenti *et al.*, 2022), BUSCO ( Manni *et al.*, 2021) and MERQURY.FK ( Rhie *et al.*, 2020). The organelle genomes were assembled using OATK ( Zhou, 2023).

***Curation***

The assembly was decontaminated using the Assembly Screen for Cobionts and Contaminants (ASCC) pipeline (article in preparation). Manual curation was primarily conducted using PretextView ( Harry, 2022), with additional insights provided by JBrowse2 ( Diesh *et al.*, 2023) and HiGlass ( Kerpedjiev *et al.*, 2018). Scaffolds were visually inspected and corrected as described by Howe *et al*. (2021). Any identified contamination, missed joins, and mis-joins were corrected, and duplicate sequences were tagged and removed. The process is documented at https://gitlab.com/wtsi-grit/rapid-curation (article in preparation).

***Evaluation of final assembly***

A Hi-C map for the final assembly was produced using bwa-mem2 ( Vasimuddin *et al.*, 2019) in the Cooler file format ( Abdennur & Mirny, 2020). To assess the assembly metrics, the *k*-mer completeness and QV consensus quality values were calculated in Merqury ( Rhie *et al.*, 2020). This work was done using the “sanger-tol/readmapping” ( Surana *et al.*, 2023a) and “sanger-tol/genomenote” ( Surana *et al.*, 2023b) pipelines. The genome readmapping pipelines were developed using the nf-core tooling ( Ewels *et al.*, 2020), use MultiQC ( Ewels *et al.*, 2016), and make extensive use of the Conda package manager, the Bioconda initiative ( Grüning *et al.*, 2018), the Biocontainers infrastructure ( da Veiga Leprevost *et al.*, 2017), and the Docker ( Merkel, 2014) and Singularity ( Kurtzer *et al.*, 2017) containerisation solutions. The genome was also analysed within the BlobToolKit environment ( Challis *et al.*, 2020) and BUSCO scores ( Manni *et al.*, 2021) were calculated.

Table 4 contains a list of relevant software tool versions and sources.

**Table 4.** Software tools: versions and sources.

Software tool	Version	Source
BlobToolKit	4.2.1	https://github.com/blobtoolkit/blobtoolkit
BUSCO	5.3.2	https://gitlab.com/ezlab/busco
bwa-mem2	2.2.1	https://github.com/bwa-mem2/bwa-mem2
Cooler	0.8.11	https://github.com/open2c/cooler
Gfastats	1.3.6	https://github.com/vgl-hub/gfastats
Hifiasm	0.16.1-r375	https://github.com/chhylp123/hifiasm
HiGlass	1.11.6	https://github.com/higlass/higlass
Merqury.FK	d00d98157618f4e8d1a9190026b19b471055b22e	https://github.com/thegenemyers/MERQURY.FK
OATK	0.1	https://github.com/c-zhou/oatk
PretextView	0.2	https://github.com/wtsi-hpag/PretextView
purge_dups	1.2.3	https://github.com/dfguan/purge_dups
sanger-tol/genomenote	v1.0	https://github.com/sanger-tol/genomenote
sanger-tol/readmapping	1.1.0	https://github.com/sanger-tol/readmapping/tree/1.1.0
Singularity	3.9.0	https://github.com/sylabs/singularity
YaHS	1.1a.2	https://github.com/c-zhou/yahs

### Wellcome Sanger Institute – Legal and Governance

The materials that have contributed to this genome note have been supplied by a Darwin Tree of Life Partner. The submission of materials by a Darwin Tree of Life Partner is subject to the **‘Darwin Tree of Life Project Sampling Code of Practice’**, which can be found in full on the Darwin Tree of Life website here. By agreeing with and signing up to the Sampling Code of Practice, the Darwin Tree of Life Partner agrees they will meet the legal and ethical requirements and standards set out within this document in respect of all samples acquired for, and supplied to, the Darwin Tree of Life Project.

Further, the Wellcome Sanger Institute employs a process whereby due diligence is carried out proportionate to the nature of the materials themselves, and the circumstances under which they have been/are to be collected and provided for use. The purpose of this is to address and mitigate any potential legal and/or ethical implications of receipt and use of the materials as part of the research project, and to ensure that in doing so we align with best practice wherever possible. The overarching areas of consideration are:

•     Ethical review of provenance and sourcing of the material

•     Legality of collection, transfer and use (national and international)

Each transfer of samples is further undertaken according to a Research Collaboration Agreement or Material Transfer Agreement entered into by the Darwin Tree of Life Partner, Genome Research Limited (operating as the Wellcome Sanger Institute), and in some circumstances other Darwin Tree of Life collaborators.

## Data Availability

European Nucleotide Archive: *Euphrasia confusa* (eyebright). Accession number PRJEB61852; https://identifiers.org/ena.embl/PRJEB61852. The genome sequence is released openly for reuse. The *Euphrasia confusa* genome sequencing initiative is part of the Darwin Tree of Life (DToL) project. All raw sequence data and the assembly have been deposited in INSDC databases. The genome will be annotated using available RNA-Seq data and presented through the Ensembl pipeline at the European Bioinformatics Institute. Raw data and assembly accession identifiers are reported in Table 1.

## Author information

Members of the Royal Botanic Garden Edinburgh Genome Acquisition Lab are listed here: https://doi.org/10.5281/zenodo.4786682.

Members of the Plant Genome Sizing collective are listed here: https://doi.org/10.5281/zenodo.7994306.

Members of the Darwin Tree of Life Barcoding collective are listed here: https://doi.org/10.5281/zenodo.12158331

Members of the Wellcome Sanger Institute Tree of Life Management, Samples and Laboratory team are listed here: https://doi.org/10.5281/zenodo.12162482.

Members of Wellcome Sanger Institute Scientific Operations: Sequencing Operations are listed here: https://doi.org/10.5281/zenodo.12165051.

Members of the Wellcome Sanger Institute Tree of Life Core Informatics team are listed here: https://doi.org/10.5281/zenodo.12160324.

Members of the Tree of Life Core Informatics collective are listed here: https://doi.org/10.5281/zenodo.12205391.

Members of the Darwin Tree of Life Consortium are listed here: https://doi.org/10.5281/zenodo.4783558.
