# Supplementary material for: The genome sequence of the confused eyebright, Euphrasia confusa Pugsley
Source: Wellcome Open Res. 2024 Nov 7;9:662. [Version 1] doi: 10.12688/wellcomeopenres.23301.1 (PMC11624436; doi:10.12688/wellcomeopenres.23301.1)
Supplement: Peer review history

# Reviewer report: Reviewer response for version 1

**DOI:** 10.21956/wellcomeopenres.25683.r113965

**Published:** 2024-12-06

**Authors:** Andrea Pedrosa-Harand

**Affiliations:** 1 Federal University of Pernambuco, Recife, PE, Brazil

The Data Note 'The genome sequence of the confused eyebright, *Euphrasia confusa* Pugsley' provide the sequencing and assembly results for a hemi-parasitic tetraploid plant. It employs high quality, state of the art methods, giving links to detailed protocols. Results are clearly presented and illustrated, suggesting an allotetraploid origin for the species, given its 22 chromosome-level scaffolds generated, instead of 11. Further annotation and synteny analyses will allow testing this hypothesis, as well as investigating other relevant aspect of this plant genome.

Are sufficient details of methods and materials provided to allow replication by others?

Yes

Is the rationale for creating the dataset(s) clearly described?

Yes

Are the datasets clearly presented in a useable and accessible format?

Yes

Are the protocols appropriate and is the work technically sound?

Yes

Reviewer Expertise:

molecular cytogenetics, plant genomics

I confirm that I have read this submission and believe that I have an appropriate level of expertise to confirm that it is of an acceptable scientific standard.

# Reviewer report: Reviewer response for version 1

**DOI:** 10.21956/wellcomeopenres.25683.r113958

**Published:** 2024-12-06

**Authors:** Deniz Aygören Uluer

**Affiliations:** 1 Ahi Evran University, Kirşehir, Turkey

This is a very clear and well written manuscript. The Introduction is sufficient for a genome sequence paper. The methods are clearly defined and easy to read. I only can suggest that instead of *Euphrasia confusa*, it is OK to use "E. confusa" after the first use. I suggest indexing.

Are sufficient details of methods and materials provided to allow replication by others?

Yes

Is the rationale for creating the dataset(s) clearly described?

Yes

Are the datasets clearly presented in a useable and accessible format?

Yes

Are the protocols appropriate and is the work technically sound?

Yes

Reviewer Expertise:

Systematic botany

I confirm that I have read this submission and believe that I have an appropriate level of expertise to confirm that it is of an acceptable scientific standard.

# Reviewer report: Reviewer response for version 1

**DOI:** 10.21956/wellcomeopenres.25683.r110720

**Published:** 2024-12-06

**Authors:** Yang Jae Kang

**Affiliations:** 1 Gyeongsang National University, Jinju-si, Gyeongsangnam-do, South Korea

The paper is overall well-written, and the methods for genome data generation and assembly are clearly described. The use of advanced sequencing technologies, such as PacBio HiFi reads and Hi-C scaffolding, is commendable and has resulted in a high-quality genome assembly. However, I would recommend including an additional validation approach to strengthen the evidence for tetraploidy. Specifically, performing a Ks (synonymous substitution rate) value distribution analysis using intraspecies synteny could help identify genomic traces of polyploidy and provide further insights into the evolutionary history of *Euphrasia confusa*. This would complement the assembly metrics and enhance the biological interpretation of the findings.

Are sufficient details of methods and materials provided to allow replication by others?

Yes

Is the rationale for creating the dataset(s) clearly described?

Yes

Are the datasets clearly presented in a useable and accessible format?

Yes

Are the protocols appropriate and is the work technically sound?

Yes

Reviewer Expertise:

plant genomics, evolution

I confirm that I have read this submission and believe that I have an appropriate level of expertise to confirm that it is of an acceptable scientific standard, however I have significant reservations, as outlined above.
